# Supplementary material for: Osteogenesis Imperfecta: Search for Mutations in Patients from the Republic of Bashkortostan (Russia)
Source: Genes (Basel). 2022 Jan 10;13(1):124. doi: 10.3390/genes13010124 (PMC8774438; doi:10.3390/genes13010124)
Supplement: Supplementary file 1 [file genes-13-00124-s001.zip › genes-1527056 supplementary.pdf]

Supplementary Table S1. Pathogenic mutations identified in the Bashkortostan Republic of Russia.

Gene	Number	dbSNP	Coding variant	Protein effect	Mutation effect	Reference
COL1A1	*1*	rs72667023	c.579delT	p. Gly194fs	deletion	Swinnen et al., 2009 [1] Zhang et al., 2011 [2] Fuccio et al., 2011 [3] Venturi et al., 2006 [4] Lindahl et al., 2015 [5] Lin et al., 2015 [6] Rolvien et al., 2018 [7] Zhytnik et al. 2019 [8] Nadyrshina et al., 2012 [9]
	2		c.2444delG	p. Gly815fs	deletion	Kataoka et al., 2007 [10] Nadyrshina et al., 2012 [9]
	3		c.375dupC	p. Ala126fs	duplication	Novel
	4		c.407dupG	p. Gly136fs		Novel
	5	rs762979302	c.358C>T	p. Arg120*	nonsense mutations	Bardai et al., 2016 [11]
	6	rs72667036	c.658C>T	p. Arg220*		Gentile et al., 2013 [12] Lin et al., 2015 [6] Zhang et al., 2015 [13] Zhang et al., 2011 [2] Körkkö et al., 1998 [14] Lindahl et al., 2015 [5]
	7		c.967G>T	p. Gly323*		Nadyrshina et al., 2012 [9]
	8	rs72645366	c.1081C>T	p. Arg361*		Körkkö et al., 1998 [14] Benušiené and Kucinskas, 2003 [15] Roschger et al., 2008 [16] Zhang et al., 2011 [2] Lindahl et al., 2015 [5] van Dijk et al., 2011 [17] Zhytnik et al., 2019 [8]

					Nadyrshina et al., 2012 [9]
9	rs72648326	c.1243C>T	p. Arg415*		Willing et al., 1996 [18]<br>Ries-Levavi et al., 2004 [19]<br>Reis et al., 2005 [20]<br>Lindahl et al., 2015 [5]<br>Hruskova et al., 2016 [21]
10	rs72653161	c.2869C>T	p. Gln957*		Ries-Levavi et al., 2004 [19]<br>Nadyrshina et al., 2012 [9]
11	rs72653173	c.3076C>T	p. Arg1026*		Ries et al., 2000 [22]<br>Ries-Levavi et al., 2004 [19]<br>Hartikka et al., 2004 [23]<br>Gentile et al., 2013 [12]<br>Zhang et al., 2011 [2]<br>Niramitmahapanya et al., 2013 [24]<br>Lin et al., 2015 [6]<br>Kaneto et al., 2014 [25]<br>Duan et al., 2016 [26]
12		c.3792delG	p. Met1264fs	Frameshift mutation	Novel
13	rs67569268	c.858+1G>A	-		Körkkö et al., 1998 [14]
14	rs72648337	c.1354-12G>A	-		Körkkö et al., 1998 [14]<br>Marini et al., 2007 [27]<br>Lindahl et al., 2015 [5]<br>Lin et al., 2015 [6]
15	rs67693970	c.2461G>A	p. Gly821Ser	missense mutation	Marini et al., 2007 [27]<br>Lund et al., 1997 [28]<br>Wang et al., 2006 [29]<br>Venturi et al., 2006 [4]<br>Lee et al., 2006 [30]<br>Zhang et al., 2011 [2]<br>Fuccio et al., 2011 [3]<br>Lin et al., 2015 [6]<br>Lindahl et al., 2015 [5]

						Kloen et al., 2018 [31]<br>Mohd Nawawi et al., 2018 [32]<br>Ho Duy et al., 2016 [33]
	16	rs72653141	c.2569G>T	p. Gly857Cys		Marini et al., 2007 [27]<br>Wang et al., 2015 [34]
COL1A2	*1*	rs756743425	c.647G>A	p. Arg216His	missense mutation	Novel
	2	rs906553840	c. 874G>A	p. Gly292Ser		Lindahl et al., 2015 [5]<br>Ho Duy et al., 2016 [33]<br>Rolvien et al., 2018 [7]
	3	rs758673298	c.1826G>A	p. Arg609Gln		Novel
	4	rs68132885	c.1197+5G>A	-		Lindahl et al., 2015 [5]<br>Marini et al., 2007 [27]<br>Nicholls et al., 1996 [35]
	5		c.2341G>C	p. Gly781Arg		Novel
	6	rs1554398261	c.2756G>A	p. Gly919Asp		Barkova et al., 2014 [36]
	7		c.2971G>C	p. Gly991Arg		Novel
	8	rs72659319	c.3034G>A	p. Gly1012Ser		Marini et al., 2007 [27]<br>Marini et al., 1993 [37]<br>Sztrolovics et al., 1993 [38]<br>Forlino et al., 1997 [39]<br>Hartikka et al., 2004 [23]<br>Lee et al., 2006 [30]<br>Lindahl et al., 2015[5]<br>Ho Duy et al., 2016 [33]<br>Stephen et al., 2015 [40]<br>Mohd Nawawi et al., 2018 [32]
	9		c.3277G>A	p. Gly1093Ser		Novel
	10		c.3977A>G	p. Lys1326Arg		Novel
	11		c.1897_1902dupGCTGGT	p. Ala633_Gly634dup	duplication	Novel
P3H1	*1*		c.1051G>T	p. Glu351*	nonsense mutation	Novel

IFITM5	*1*	rs587776916	c.-14C>T	-	start codon mutation	Semler et al., 2012 [41]<br>Balasubramanian et al., 2013 [42]<br>Cho et al., 2012 [43]<br>Grover et al., 2013 [44]<br>Takagi et al., 2013 [45]<br>Kim et al., 2013 [46]<br>Zhang et al., 2013 [47]<br>Guillén-Navarro et al., 2014 [48]<br>Rauch et al., 2014 [49]<br>Lazarus et al., 2014 [50]

References

1. Swinnen, F.K.; De Leenheer, E.M.; Coucke, P.J.; Cremers, C.W.; Dhooge, I.J. Audiometric, surgical, and genetic findings in 15 ears of patients with osteogenesis imperfecta. *Laryngoscope* **2009**, *119*, 1171–1179. https://doi.org/10.1002/lary.20155.
2. Zhang, Z.-L.; Zhang, H.; Ke, Y.-H.; Yue, H.; Xiao, W.-J.; Yu, J.-B.; Gu, J.-M.; Hu, W.-W.; Wang, C.; He, J.-W.; et al. The identification of novel mutations in COL1A1, COL1A2, and LEPRE1 genes in Chinese patients with osteogenesis imperfecta. *J. Bone Miner. Metab.* **2012**, *30*, 69–77. https://doi.org/10.1007/s00774-011-0284-6.
3. Fuccio, A.; Iorio, M.; Amato, F.; Elce, A.; Ingino, R.; Filocamo, M.; Castaldo, G.; Salvatore, F.; Tomaiuolo, R. A Novel DHPLC-Based Procedure for the Analysis of COL1A1 and COL1A2 Mutations in Osteogenesis Imperfecta. *J. Mol. Diagn.* **2011**, *13*, 648–656. https://doi.org/10.1016/j.jmoldx.2011.06.006.
4. Venturi, G.; Tedeschi, E.; Mottes, M.; Valli, M.; Camilot, M.; Viglio, S.; Antoniazzi, F.; Tatò, L. Osteogenesis imperfecta: Clinical, biochemical and molecular findings. *Clin. Genet.* **2006**, *70*, 131–139. https://doi.org/10.1111/j.1399-0004.2006.00646.x.
5. Lindahl, K.; Åström, E.; Rubin, C.-J.; Grigelioniene, G.; Malmgren, B.; Ljunggren, Ö.; Kindmark, A. Genetic epidemiology, prevalence, and genotype–phenotype correlations in the Swedish population with osteogenesis imperfecta. *Eur. J. Hum. Genet.* **2015**, *23*, 1042–1050. https://doi.org/10.1038/ejhg.2015.81.
6. Lin, H.-Y.; Chuang, C.-K.; Su, Y.-N.; Chen, M.-R.; Chiu, H.-C.; Niu, D.-M.; Lin, S.-P. Genotype and phenotype analysis of Taiwanese patients with osteogenesis imperfecta. *Orphanet J. Rare Dis.* **2015**, *10*, 152. https://doi.org/10.1186/s13023-015-0370-2.
7. Rolvien, T.; Stürznickel, J.; Schmidt, F.N.; Butscheidt, S.; Schmidt, T.; Busse, B.; Mundlos, S.; Schinke, T.; Kornak, U.; Amling, M.; et al. Comparison of Bone Microarchitecture Between Adult Osteogenesis Imperfecta and Early-Onset Osteoporosis. *Calcif. Tissue Int.* **2018**, *103*, 512–521. https://doi.org/10.1007/s00223-018-0447-8.
8. Zhytnik, L.; Maasalu, K.; Pashenko, A.; Khmyzov, S.; Reimann, E.; Prans, E.; Kõks, S.; Märtson, A. COL1A1/2 Pathogenic Variants and Phenotype Characteristics in Ukrainian Osteogenesis Imperfecta Patients. *Front. Genet.* **2019**, *10*, 722. https://doi.org/10.3389/fgene.2019.00722.
9. Nadyrshina, D.D.; Khusainova, R.I.; Khusnutdinova, E.K. Studies of type I collagen (COL1A1) alpha1 chain in patients with osteogenesis imperfecta. *Genetika* **2012**, 48, 372–380. Russian. PMID: 22679784.
10. Kataoka, K.; Ogura, E.; Hasegawa, K.; Inoue, M.; Seino, Y.; Morishima, T.; Tanaka, H. Mutations in type I collagen genes in Japanese osteogenesis imperfecta patients. *Pediatr. Int.* **2007**, *49*, 564–569. https://doi.org/10.1111/j.1442-200x.2007.02422.x.
11. Bardai, G.; Moffatt, P.; Glorieux, F.H.; Rauch, F. DNA sequence analysis in 598 individuals with a clinical diagnosis of osteogenesis imperfecta: Diagnostic yield and mutation spectrum. *Osteoporos. Int.* **2016**, *27*, 3607–3613. https://doi.org/10.1007/s00198-016-3709-1.
12. Gentile, F.V.; Zuntini, M.; Parra, A.; Battistelli, L.; Pandolfi, M.; Pals, G.; Sangiorgi, L. Validation of a quantitative PCR-high-resolution melting protocol for simultaneous screening ofCOL1A1andCOL1A2point mutations and large rearrangements: Application for diagnosis of osteogenesis imperfecta. *Hum. Mutat.* **2012**, *33*, 1697–1707. https://doi.org/10.1002/humu.22146.

13. Zhang, H.; Yang, R.; Wang, Y.; Ye, J.; Han, L.; Qiu, W.; Gu, X. A pilot study of gene testing of genetic bone dysplasia using targeted next-generation sequencing. *J. Hum. Genet.* **2015**, *60*, 769–776. https://doi.org/10.1038/jhg.2015.112.
14. Körkkö, J.; Ala-Kokko, L.; De Paepe, A.; Nuytinck, L.; Earley, J.; Prockop, D.J. Analysis of the COL1A1 and COL1A2 Genes by PCR Amplification and Scanning by Conformation-Sensitive Gel Electrophoresis Identifies Only COL1A1 Mutations in 15 Patients with Osteogenesis Imperfecta Type I: Identification of Common Sequences of Null-Allele Mutations. *Am. J. Hum. Genet.* **1998**, *62*, 98–110. https://doi.org/10.1086/301689.
15. Benusiené, E.; Kucinskas, V. COL1A1 mutation analysis in Lithuanian patients with osteogenesis imperfecta. *J. Appl. Genet.* **2003**, *44*, 95–102.
16. Roschger, P.; Fratzl-Zelman, N.; Misof, B.M.; Glorieux, F.H.; Klaushofer, K.; Rauch, F. Evidence that Abnormal High Bone Mineralization in Growing Children with Osteogenesis Imperfecta is not Associated with Specific Collagen Mutations. *Calcif. Tissue Int.* **2008**, *82*, 263–270. https://doi.org/10.1007/s00223-008-9113-x.
17. Van Dijk, F.; Cobben, J.; Kariminejad, A.; Maugeri, A.; Nikkels, P.; van Rijn, R.; Pals, G. Osteogenesis Imperfecta: A Review with Clinical Examples. *Mol. Syndr.* **2011**, *2*, 1–20. https://doi.org/10.1159/000332228.
18. Willing, M.C.; Deschenes, S.P.; Slayton, R.L.; Roberts, E.J. Premature chain termination is a unifying mechanism for COL1A1 null alleles in osteogenesis imperfecta type I cell strains. *Am. J. Hum. Genet.* **1996**, *59*, 799–809.
19. Ries-Levavi, L.; Ish-Shalom, T.; Frydman, M.; Lev, D.; Cohen, S.; Barkai, G.; Goldman, B.; Byers, P.; Friedman, E. Genetic and biochemical analyses of Israeli osteogenesis imperfecta patients. *Hum. Mutat.* **2004**, *23*, 399–400. https://doi.org/10.1002/humu.9230.
20. Reis, F.C.; Alexandrino, F.; E Steiner, C.; Norato, D.Y.J.; Cavalcanti, D.P.; Sartorato, E.L. Molecular findings in Brazilian patients with osteogenesis imperfecta. *J. Appl. Genet.* **2005**, *46*, 105–108.
21. Hruskova, L.; Fijalkowski, I.; Van Hul, W.; Marik, I.; Mortier, G.; Martasek, P.; Mazura, I. Eight mutations including 5 novel ones in the COL1A1 gene in Czech patients with osteogenesis imperfecta. *Biomed. Pap.* **2016**, *160*, 442–447. https://doi.org/10.5507/bp.2016.022.
22. Ries, L.; Frydman, M.; Barkai, G.; Goldman, B.; Friedman, E. Prenatal diagnosis of a novelCOL1A1 mutation in osteogenesis imperfecta type I carried through full term pregnancy. *Prenat. Diagn.* **2000**, *20*, 876–880. https://doi.org/10.1002/1097-0223(200011)20:113.0.co;2-x.
23. Hartikka, H.; Kuurila, K.; Körkkö, J.; Kaitila, I.; Grénman, R.; Pynnönen, S.; Hyland, J.C.; Ala-Kokko, L. Lack of correlation between the type ofCOL1A1orCOL1A2mutation and hearing loss in osteogenesis imperfecta patients. *Hum. Mutat.* **2004**, *24*, 147–154. https://doi.org/10.1002/humu.20071.
24. Niramitmahapanya, S.; Anusornvongchai, T.; Pingsuthiwong, S.; Sarinnapakorn, V.; Deerochanawong, C.; Sunthornthepvarakul, T. Novel COL1A1 gene mutation (R1026X) of type I osteogenesis imperfecta: A first case report. *J. Med. Assoc. Thail. = Chotmaihet thangphaet* **2013**, *96*, 23682531.
25. Kaneto, C.M.; Lima, P.S.; Zanette, D.L.; Prata, K.L.; Neto, J.M.P.; de Paula, F.J.; A Silva, W. COL1A1and miR-29b show lower expression levels during osteoblast differentiation of bone marrow stromal cells from Osteogenesis Imperfecta patients. *BMC Med Genet.* **2014**, *15*, 45. https://doi.org/10.1186/1471-2350-15-45.
26. Duan, H.; Yan, Z.; Lu, Y.; Cheng, J.; Zhang, D.; Yuan, H.; Han, D. Identification of two recurrent mutations of COL1A1 gene in Chinese Van der Hoeve syndrome patients. *Acta Oto-Laryngol.* **2016**, *136*, 786–791. https://doi.org/10.3109/00016489.2016.1159327.
27. Marini, J.C.; Forlino, A.; Cabral, W.A.; Barnes, A.; Antonio, J.D.S.; Milgrom, S.; Hyland, J.C.; Körkkö, J.; Prockop, D.J.; De Paepe, A.; et al. Consortium for osteogenesis imperfecta mutations in the helical domain of type I collagen: Regions rich in lethal mutations align with collagen binding sites for integrins and proteoglycans. *Hum. Mutat.* **2007**, *28*, 209–221. https://doi.org/10.1002/humu.20429
28. Lund, A.M.; Skovby, F.; Schwartz, M. Serine for glycine substitutions in the C-terminal third of the alpha 1(I) chain of collagen I in five patients with nonlethal osteogenesis imperfecta. *Hum Mutat.* **1997**, *9*, 378–382. https://doi.org/10.1002/(SICI)1098-1004(1997)9:43.0.CO;2-#. PMID: 9101304. .
29. Wang, Z.; Xu, D.-L.; Hu, J.-Y.; Liao, Y.-H.; Yang, Z.; Liang, Q.; Wang, L.-T. Gene mutation analysis of a Chinese family with osteogenesis imperfecta. *Zhonghua Yi Xue Yi Chuan Xue Za Zhi* **2006**, *23*, 192–194.
30. Lee, K.-S.; Song, H.-R.; Cho, T.-J.; Kim, H.J.; Lee, T.-M.; Jin, H.-S.; Park, H.-Y.; Kang, S.; Jung, S.-C.; Koo, S.K. Mutational spectrum of type I collagen genes in Korean patients with osteogenesis imperfecta. *Hum. Mutat.* **2006**, *27*, 599. https://doi.org/10.1002/humu.9423.
31. Kloen, P.; Donders, J.C.; Eekhoff, E.M.W.; Hamdy, R.C. Pauwels Osteotomy for Femoral Neck Nonunion in Two Adult Siblings with Osteogenesis Imperfecta. *Hip Pelvis* **2018**, *30*, 53–59. https://doi.org/10.5371/hp.2018.30.1.53.
32. Nawawi, N.M.; Selveindran, N.M.; Rasat, R.; Chow, Y.P.; Latiff, Z.A.; Zakaria, S.Z.S.; Jamal, R.; Murad, N.A.A.; Aziz, B.B.A. Genotype-phenotype correlation among Malaysian patients with osteogenesis imperfecta. *Clin. Chim. Acta* **2018**, *484*, 141–147. https://doi.org/10.1016/j.cca.2018.05.048.

33. Duy, B.H.; Zhytnik, L.; Maasalu, K.; Kändla, I.; Prans, E.; Reimann, E.; Märtson, A.; Kõks, S. Mutation analysis of the COL1A1 and COL1A2 genes in Vietnamese patients with osteogenesis imperfecta. *Hum. Genom.* **2016**, *10*, 27. https://doi.org/10.1186/s40246-016-0083-1.
34. Wang, Y.; Cui, Y.; Zhou, X.; Han, J. Development of a High-Throughput Resequencing Array for the Detection of Pathogenic Mutations in Osteogenesis Imperfecta. *PLoS ONE* **2015**, *10*, e0119553. https://doi.org/10.1371/journal.pone.0119553.
35. Nicholls, A.C.; Oliver, J.; McCarron, S.; Winter, G.B.; Pope, F.M. Splice site mutation causing deletion of exon 21 sequences from the pro alpha 2(I) chain of type I collagen in a patient with severe dentinogenesis imperfecta but very mild osteogenesis imper-fecta. *Hum. Mutat.* **1996**, *7*, 219–227. https://doi.org/10.1002/(SICI)1098-1004(1996)7:33.0.CO;2-5. PMID: 8829655.
36. Barkova, E.; Mohan, U.; Chitayat, D.; Keating, S.; Toi, A.; Frank, J.; Frank, R.; Tomlinson, G.; Glanc, P. Fetal skeletal dysplasias in a tertiary care center: Radiology, pathology, and molecular analysis of 112 cases. *Clin. Genet.* **2014**, *87*, 330–337. https://doi.org/10.1111/cge.12434.
37. Marini, J.C.; Lewis, M.B.; Wang, Q.; Chen, K.J.; Orrison, B.M. Serine for glycine substitutions in type I collagen in two cases of type IV osteogenesis imperfecta (OI). Additional evidence for a regional model of OI pathophysiology. *J. Biol. Chem.* **1993**, *268*, 2667–2673.
38. Sztrolovics, R.; Glorieux, F.; Van Der Rest, M.; Roughley, P. Identification of type I collagen gene (COL1A2) mutations in nonlethal osteogenesis imperfecta. *Hum. Mol. Genet.* **1993**, *2*, 1319–1321. https://doi.org/10.1093/hmg/2.8.1319.
39. Forlino, A.; D'Amato, E.; Valli, M.; Camera, G.; Hopkins, E.; Marini, J.C.; Cetta, G.; Coviello, D. Phenotypic Comparison of an Osteogenesis Imperfecta Type IV Proband with a de Novoα2(I) Gly922 → Ser Substitution in Type I Collagen and an Unrelated Patient with an Identical Mutation. *Biochem. Mol. Med.* **1997**, *62*, 26–35. https://doi.org/10.1006/bmme.1997.2620.
40. Stephen, J.; Girisha, K.M.; Dalal, A.; Shukla, A.; Shah, H.; Srivastava, P.; Kornak, U.; Phadke, S.R. Mutations in patients with osteogenesis imperfecta from consanguineous Indian families. *Eur. J. Med. Genet.* **2015**, *58*, 21–27. https://doi.org/10.1016/j.ejmg.2014.10.001.
41. Semler, O.; Garbes, L.; Keupp, K.; Swan, D.; Zimmermann, K.; Becker, J.; Iden, S.; Wirth, B.; Eysel, P.; Koerber, F.; et al. A Mutation in the 5′-UTR of IFITM5 Creates an In-Frame Start Codon and Causes Autosomal-Dominant Osteogenesis Imperfecta Type V with Hyperplastic Callus. *Am. J. Hum. Genet.* **2012**, *91*, 349–357. https://doi.org/10.1016/j.ajhg.2012.06.011.
42. Balasubramanian, M.; Parker, M.J.; Dalton, A.; Giunta, C.; Lindert, U.; Peres, L.C.; Wagner, B.E.; Arundel, P.; Offiah, A.; Bishop, N.J. Genotype–phenotype study in type V osteogenesis imperfecta. *Clin. Dysmorphol.* **2013**, *22*, 93–101. https://doi.org/10.1097/mcd.0b013e32836032f0.
43. Cho, T.-J.; Lee, K.-E.; Lee, S.-K.; Song, S.J.; Kim, K.J.; Jeon, D.; Lee, G.; Kim, H.-N.; Lee, H.R.; Eom, H.-H.; et al. A Single Recurrent Mutation in the 5′-UTR of IFITM5 Causes Osteogenesis Imperfecta Type V. *Am. J. Hum. Genet.* **2012**, *91*, 343–348. https://doi.org/10.1016/j.ajhg.2012.06.005.
44. Grover, M.; Campeau, P.M.; Lietman, C.D.; Lu, J.T.; Gibbs, R.A.; Schlesinger, A.E.; Lee, B.H. Osteogenesis imperfecta without features of type V caused by a mutation in the IFITM5 gene. *J. Bone Miner. Res.* **2013**, *28*, 2333–2337. https://doi.org/10.1002/jbmr.1983.
45. Takagi, M.; Sato, S.; Hara, K.; Tani, C.; Miyazaki, O.; Nishimura, G.; Hasegawa, T. A recurrent mutation in the 5′-UTR of IFITM5 causes osteogenesis imperfecta type V. *Am. J. Med. Genet. Part A* **2013**, *161*, 1980–1982. https://doi.org/10.1002/ajmg.a.36025.
46. Kim, O.-H.; Jin, D.-K.; Kosaki, K.; Kim, J.-W.; Cho, S.Y.; Yoo, W.J.; Choi, I.H.; Nishimura, G.; Ikegawa, S.; Cho, T.-J. Osteogenesis imperfecta type V: Clinical and radiographic manifestations in mutation confirmed patients. *Am. J. Med. Genet. Part A* **2013**, *161*, 1972–1979. https://doi.org/10.1002/ajmg.a.36024.
47. Zhang, Z.; Li, M.; He, J.-W.; Fu, W.-Z.; Zhang, C.-Q.; Zhang, Z.-L. Phenotype and Genotype Analysis of Chinese Patients with Osteogenesis Imperfecta Type V. *PLoS ONE* **2013**, *8*, e72337. https://doi.org/10.1371/journal.pone.0072337.
48. Guillén-Navarro, E.; Ballesta-Martínez, M.J.; Valencia, M.; Bueno, A.M.; Martinez-Glez, V.; López-González, V.; Burnyte, B.; Utkus, A.; Lapunzina, P.; Ruiz-Perez, V.L. Two mutations inIFITM5causing distinct forms of osteogenesis imperfecta. *Am. J. Med. Genet. Part A* **2014**, *164*, 1136–1142. https://doi.org/10.1002/ajmg.a.36409.
49. Rauch, F.; Lalic, L.; Glorieux, F.H.; Moffatt, P.; Roughley, P. Targeted Sequencing of a Pediatric Metabolic Bone Gene Panel Using a Desktop Semiconductor Next-Generation Sequencer. *Calcif. Tissue Res.* **2014**, *95*, 323–331. https://doi.org/10.1007/s00223-014-9897-9.
50. Lazarus, S.; McInerney-Leo, A.M.; McKenzie, F.A.; Baynam, G.; Broley, S.; Cavan, B.; Munns, C.F.; Pruijs, J.E.; Sillence, D.; Terhal, P.A.; et al. The IFITM5 mutation c.-14C> T results in an elongated transcript expressed in human bone; and causes varying phenotypic severity of osteogenesis imperfecta type V. *BMC Musculoskelet Disord*. **2014**, *15*, 107. https://doi.org/10.1186/1471-2474-15-107. PMID: 24674092; PMCID: PMC3986707.
